# Correction to miR‐10b‐5p rescues leaky gut linked with gastrointestinal dysmotility and diabetes

**DOI:** 10.1002/ueg2.12529

**Published:** 2024-01-05

Zogg H, Singh R, Ha SE, Wang Z, Jin B, Ha M, et al. miR‐10b‐5p rescues leaky gut linked with gastrointestinal dysmotility and diabetes. United European Gastroenterol J. 2023;11(8):750–766. 10.1002/ueg2.12463.

The *Y*‐axis values of blood glucose (mg/dL), “0, 10, 20, 30, and 40” in Figure (2b) were corrected to “0, 50, 100, 150, and 200”. The revised figure is given below:

**FIGURE 2:**
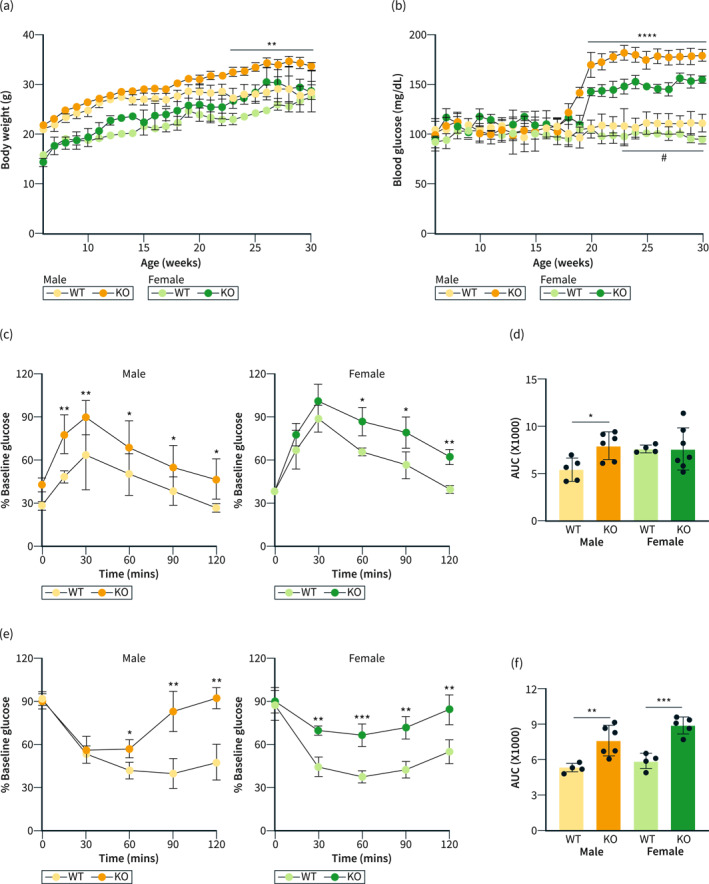


We apologize for this error.

